# Compound heterozygous mutations in *MICU1* cause myopathy with extrapyramidal signs in two Chinese pedigrees

**DOI:** 10.3389/fneur.2025.1624830

**Published:** 2025-09-12

**Authors:** Jieling Li, Hu Liu, Xiaoming Gan, Yuexu Ou, Yuanhui Duan, Jie Cao

**Affiliations:** Department of Medical General Ward, Children’s Hospital of Chongqing Medical University, National Clinical Research Center for Child Health and Disorders, Ministry of Education Key Laboratory of Child Development and Disorders, China International Science and Technology Cooperation Base of Child Development and Critical Disorders, Chongqing Key Laboratory of Child Neurodevelopment and Cognitive Disorders, Chongqing, China

**Keywords:** *MICU1*, myopathy with extrapyramidal signs, compound heterozygous mutations, clinical features, two Chinese pedigrees

## Abstract

**Background:**

Although *MICU1*-related myopathy with extrapyramidal signs (MPXPS) has been reported globally, its genotypic and phenotypic spectrum in Chinese populations remains poorly characterized. Here we investigate two unrelated Chinese pedigrees with MPXPS caused by novel compound heterozygous *MICU1* mutations, addressing this critical knowledge gap.

**Methods:**

We retrospectively analyzed the clinical features of four children from two unrelated families with MPXPS caused by compound heterozygous mutations in the *MICU1* gene. Whole exome sequencing (WES) was performed on the probands and their parents. Sanger sequencing was used to validate the candidate gene variants. A literature review and summary of cases with bi-allelic mutations in *MICU1* leading to MPXPS were conducted.

**Results:**

Four children from two unrelated families presented with elevated muscle enzymes and liver function abnormalities. In Family 1, the proband (older brother, 8 years) exhibited typical MPXPS symptoms including motor dysfunction and cognitive impairment, while his younger brother (4 years) remained asymptomatic though with elevated muscle enzymes. WES identified compound heterozygous variants c.156G > A and c.235G > T in the two siblings. In Family 2, the proband (older sister, 3 years 4 months) manifested early signs including pes planus and attention deficits, whereas her younger sister (1 year 6 months) showed no clinical manifestations despite biochemical abnormalities. WES identified compound heterozygous variants EXON4-8 heterozygous deletion and c.1,372C > T.

**Conclusion:**

The phenotypic variations between the sibling pairs across both pedigrees may indicate age-dependent disease progression. The four children in this study with MPXPS due to compound heterozygous mutations in the *MICU1* gene showed phenotypic differences compared to previously reported MPXPS cases, indicating a positive correlation between *MICU1* loss of function and the severity of the phenotype, demonstrating a clear genotype–phenotype correlation.

## Introduction

The protein encoded by the *MICU1* gene, mitochondrial calcium uptake one, plays a crucial role in cellular, particularly mitochondrial calcium homeostasis. (1) Bi-allelic mutations in *MICU1* can lead to myopathy with extrapyramidal signs (MPXPS, MIM: #615673). MPXPS is an autosomal recessive disorder characterized by early childhood onset of proximal muscle weakness and learning disabilities. While the muscle weakness is static, most patients develop progressive extrapyramidal signs that may become disabling. (2) Although several cases of MPXPS caused by bi-allelic mutations in *MICU1* have been reported abroad, the reported gene mutation sites and corresponding clinical phenotypes are limited.

This study reports the gene mutation sites and corresponding clinical features of four children with MPXPS caused by compound heterozygous mutations in the *MICU1* gene in two families, and identifies new compound heterozygous gene mutation sites and corresponding clinical phenotypes for this disease.

## Methods

The parents of the four children have read and signed the informed consent form. This study was approved by the Institutional Review Board of Children’s Hospital of Chongqing Medical University. Clinical data collected from the children included general information, physical examination, muscle enzyme profile, liver and kidney function, electrolytes, lactate, ammonia, ceruloplasmin, abdominal ultrasound, chest X-ray, electrocardiogram, echocardiogram, bilateral lower limb magnetic resonance scanning, and muscle biopsy.

Given that the four children from the two families exhibited elevated muscle enzymes and liver function abnormalities, genetic testing was conducted after medical ethics review and consent from the children’s parents. Blood samples of 5 mL were drawn from each of the four children and their parents for whole exome sequencing of the four family members. The extracted DNA samples were subjected to quality controlling using Qubit 2.0 fluorimeter and electrophoresis with 0.8% agarose gel for further protocol. Protein-coding exome enrichment was performed using xGen Exome Research Panel v1.0(IDT, Iowa, United States) that consists of 429,826 individually synthesized and quality-controlled probes, which targets 39 Mb protein-coding region (19,396 genes) of the human genome and covers 51 Mb of end-to-end tiled probe space. High-throughput sequencing was performed on Illumina NovaSeq 6,000 series sequencer (PE150), and not less than 99% of target sequence were sequenced.

## Results

The two siblings in Family 1 had unremarkable maternal and birth histories, with non-consanguineous parents who showed no clinical manifestations of MPXPS.

Child 1 (the older brother) is currently 8 years old and exhibited difficulty walking as a toddler, preference for walking on tiptoes, difficulty squatting and standing, poor balance, and inability to hop on one foot. At 6 years of age, he developed attention deficit hyperactivity disorder and learning difficulties, with a Wechsler intelligence score of 70. Neurological examination: Hip flexors/knee extensors/ankle dorsiflexors: Grade 4 + bilaterally (Medical Research Council, MRC 0–5 scale), and poor performance in hopping on one foot, able to walk on tiptoes/heels, normal muscle tone, calf hypertrophy, suspicious positive for Gower’s sign, no scoliosis, and negative pyramidal tract signs. Multiple liver function and muscle enzyme profiles: Alanine aminotransferase 104–120 U/L, Aspartate aminotransferase 104–131 U/L, Lactate dehydrogenase 564–710 U/L, Creatine kinase 6,226–7,804 U/L, Myoglobin 206.56–396.06 ug/L, Creatine kinase isoenzyme 41.64–56.62 ug/L; Electrocardiogram: Sinus arrhythmia, QT prolongation, high QT values; Bilateral lower limb magnetic resonance scanning: Changes at the distal metaphysis of the right femur, possibly a benign lesion, possibly a fibrous cortical defect. Muscle biopsy pathology: Muscle fibers are relatively well-arranged, with a predominance of type I muscle fibers, no clear characteristic pathological changes. Electrolytes, lactate, ammonia, echocardiogram, chest X-ray, and abdominal ultrasound are all normal.

Child 2 (the younger brother) is currently 4 years old, with normal motor and intellectual development, no clinical manifestations, normal neurological examination, liver function, and muscle enzyme profiles: Alanine aminotransferase 122 U/L, Aspartate aminotransferase 159 U/L, Lactate dehydrogenase 901 U/L, Creatine kinase 12,418 U/L, Myoglobin 318.15 ug/L, Creatine kinase isoenzyme 88.86 ug/L. Lactate + ammonia, abdominal ultrasound, electrocardiogram, echocardiogram, and chest X-ray are all normal.

**Figure 1:**
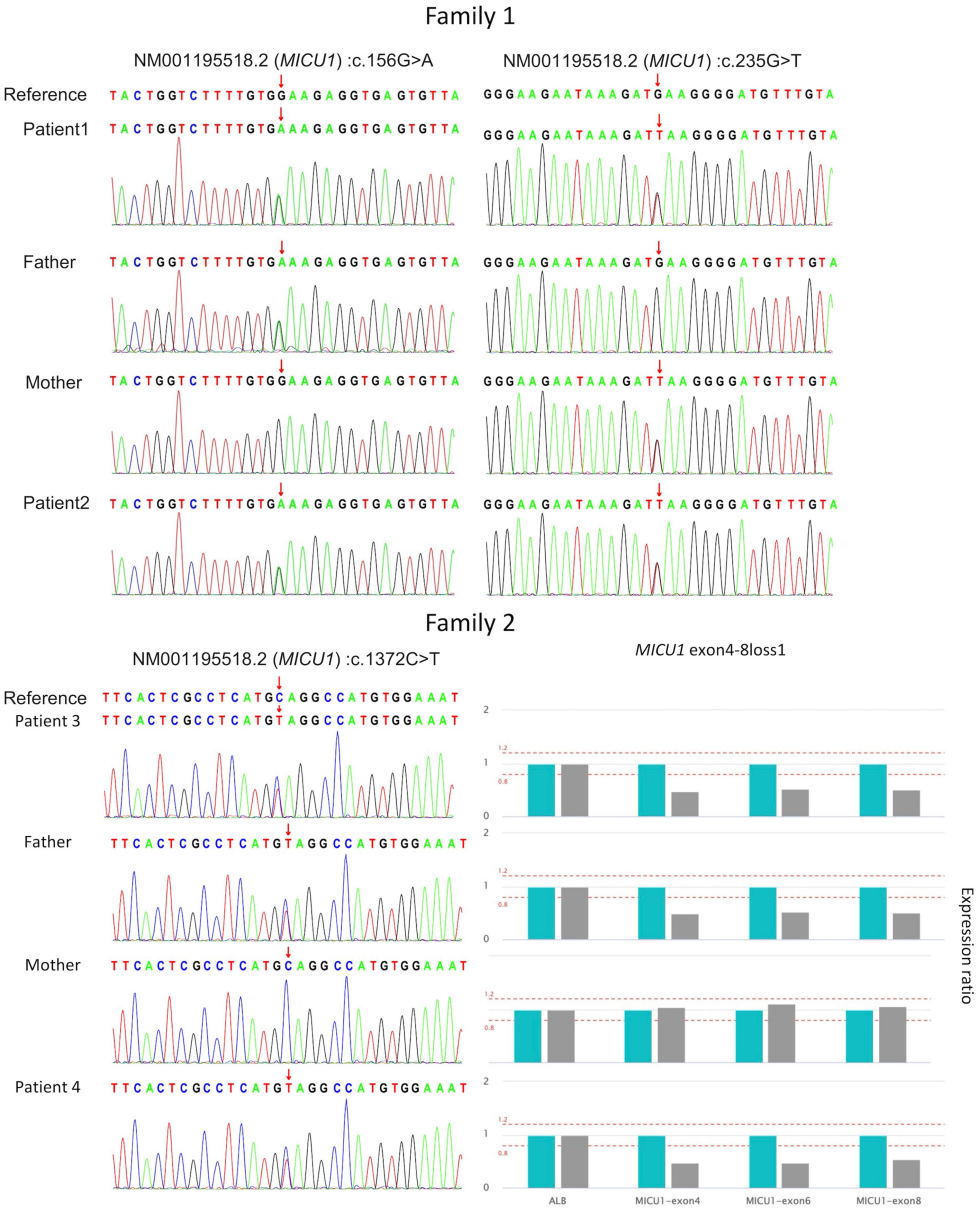
Validation of four *MICU1* gene variants in two families through Sanger sequencing and qPCR.

Genetic testing results showed compound heterozygous variants in the children’s *MICU1* gene: paternally derived c.156G > A and maternally derived c.235G > T (Figure 1), the NGS sequencing depth of the two point mutations is shown in Table 1. According to the ACMG clinical practice guidelines, the *MICU1* gene c.156G > A is pathogenic (PVS1 + PM2_Supporting+MP3), and c.235G > T is likely pathogenic (PVS1 + PM2_Supporting).

**Table 1:** Sequencing depth of three detected MICU1 point mutations in two families.

		*MICU1* variants	Sequencing depth (X)	Variant depth (X) / ratio	Variant genotype
Family 1	Patient 1	c.156G > A	325	173 / 0.5323	Heterozygous
	Father	c.156G > A	127	65 / 0.5118	Heterozygous
	Mother	c.156G > A	112	0 / 0	Wildtype
	Patient 2	c.156G > A	155	70 / 0.4516	Heterozygous
	Patient 1	c.235G > T	307	143 / 0.4658	Heterozygous
	Father	c.235G > T	151	0 / 0	Wildtype
	Mother	c.235G > T	139	57 / 0.4101	Heterozygous
	Patient 2	c.235G > T	185	95 / 0.5135	Heterozygous
Family 2	Patient 3	c.1372C > T	406	217 / 0.5345	Heterozygous
	Father	c.1372C > T	226	100 / 0.4425	Heterozygous
	Mother	c.1372C > T	180	0 / 0	Wildtype
	Patient 4	c.1372C > T	188	98 / 0.5213	Heterozygous

The two siblings in Family 2 had healthy parents, non-consanguineous marriage, and no relevant family history.

Child 3 (the older sister) is currently 3 years and 4 months old, and was found to have flat feet at 2 years of age, occasional retrosternal pain at 3 years of age, and hyperactivity and poor concentration, but normal motor function. Neurological examination was normal, with outward flaring of both feet, collapsed arches, and good activity. Multiple liver function and muscle enzyme profiles: Alanine aminotransferase 54–60 U/L, Aspartate aminotransferase 70–79 U/L, Lactate dehydrogenase 402–469 U/L, Creatine kinase 4,478–4,539 U/L, Myoglobin 70.21–84.24 ug/L, Creatine kinase isoenzyme 22.63–31.85 ug/L, electrocardiogram, echocardiogram, and chest X-ray were all normal.

Child 4 (the younger sister) is currently 1 year and 6 months old, with normal intellectual and motor development, no clinical manifestations, normal neurological examination, liver function, and muscle enzyme profiles: Alanine aminotransferase 51 U/L, Aspartate aminotransferase 60 U/L, Lactate dehydrogenase 393 U/L, Creatine kinase 1,143 U/L, Myoglobin 55.22 ug/L, Creatine kinase isoenzyme 15.67 ug/L.

**Figure 2:**
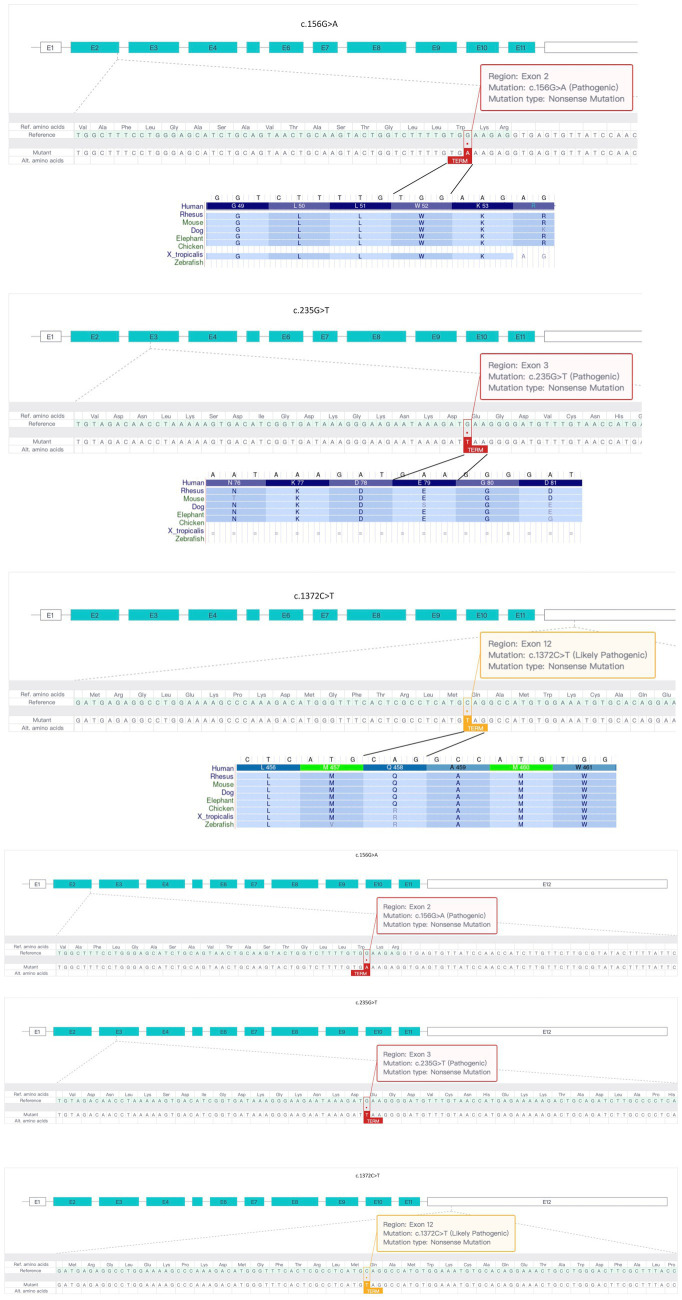
Analysis of three *MICU1* gene point mutations identified in two families. All three point mutations are nonsense mutations, but there are differences in evolutionary conservation among different species.

Genetic testing results showed that both children in Family 2 had compound heterozygous variants in the *MICU1* gene, paternally derived c.1372C > T and maternally derived EXON4-8 loss (Figure 1), the former’s NGS sequencing depth is shown in Table 1, and the latter was validated by relative quantitative PCR (qPCR). According to the ACMG clinical practice guidelines, the two variants are of uncertain pathogenicity (PVS1_Moderate+PM2_Supporting+MP3) and likely pathogenic (PVS1 + PM2_Supporting). Based on the consequences analysis of the three point mutations, c.156G > A, c.235G > T, and c.1,372C > T, identified in this study causing p. W52*, p. E79*, and p. Q458*, respectively, all are nonsense mutations, but the evolutionary conservation analysis shows different levels of conservation for the three mutations (Figure 2).

This article compares the four children from the two families with the reported families and individual cases of *MICU1* gene mutations and corresponding clinical manifestations (Table 2).

**Table 2:** The four children in this article and the reported families and individual cases of *MICU1* gene mutations and corresponding clinical manifestations.

Mutation’s number	Variant 1	Variant 2	Age	Clinical phenotypes (Number of affected/total number)					Auxiliary examination					References
				Proximal muscle weakness	Motor disorders	Learning difficulties	Extrapyramidal signs	Other symptoms and signs	Creatine kinase	Liver transaminases	Muscle biopsy	Brain/thigh MRI	其他	
1	c.1078-1(intron9)G>C	c.1078-1(intron9)G>C	11 m ~ 8y	15/15	15/15	15/15	10/15	Ataxia,microcephaly, ophthalmoplegia, ptosis, optic atrophy and axonal peripheral neuropathy	Elevated	—	Shows myopathic changes, rare necrotic fibers, and normal mitochondrial respiratory chain enzymes	—	—	(2)
2	c.741 + 1 (intron7)G>A	c.741 + 1 (intron7)G>A												
3	c.553(exon6)C>T	c.553(exon6)C>T	—	13/13	13/13	13/13	—	Failure to thrive (4/12), hypotonia (6/12), frequent falls (4/12), facial dysmorphism (3/12), hepatomegaly (3/12), and painful muscle spasms (4/12)	Elevated	Elevated	—	—	—	(4)
4	c.553(exon6)C>T	EX9-10DUP	10y					Developmental delay, ventricular septal defect, facial dysmorphism, and hypotonia			Lipid storage myopathy and normal mitochondrial complexes I to IV	Increased signal intensity in the white matter along the occipital horns of the lateral ventricles	Autoimmune hemolytic anemia, low levels of IgG, B and T cells, absence of NK cells, and pancytopenia	
5	c.161 + 1 (intron2)G>A	c.386(exon4)G>C	12y	1/1	1/1	—	1/1	Developmental delay, facial dysmorphism, ataxia, gait rigidity, seizure episodes, hyperreflexia, and amblyopia	Elevated	Elevated	—	Multifocal brain abnormalities, chronic encephalomalacia	—	(3)
6	c.52(exon1)C>T	c.553(exon6)C > T	—	1/1	1/1	1/1	—	Ataxia, developmental delay, clinodactyly, and sleep disturbances	Elevated	—	Dystrophy, neurogenic atrophy, severe mitochondrial disarray, alterations in Golgi apparatus structure, vacuolation, and lipid metabolism disorder	—	—	(5)
7	loss1(EXON: 2)	c.553(exon6)C > T	—	1/1	1/1	—	—	Bilateral calf muscle hypertrophy, ataxia, and impaired cognitive pain perception	Elevated	—		—	—	
8	c.156(exon2)G > A	c.235(exon3)G > T	8y	1/1	1/1	1/1	none	Attention Deficit Hyperactivity Disorder, calf hypertrophy, and suspicious positive for Gower’s sign	Elevated	Elevated	The muscle fibers are relatively well-organized, with a predominance of type I muscle fibers, and no clear characteristic pathological changes are observed	Changes at the distal metaphysis of the right femur, possibly indicating a benign lesion, with potential for fibrous cortical defect	Electrocardiogram: Sinus arrhythmia, prolonged QT interval, and high QT values	This study, Family 1
			4y	none	none	none	none	none			—	—	—	
9	loss1(EXON: 4–8)	c.1372(exon12)C > T	3y4m	none	none	1/1	none	Flat feet, retrosternal pain, hyperactivity, poor concentration, outward flaring of both feet, and collapsed arches of both feet	Elevated	Elevated	—	—	—	This study , Family 2
			1y6m	none	none	none	none	none	Elevated	Elevated	—	—	—	

none, indicates not detected; —, indicates unknown or not mentioned in the literature.

## Discussion

Mutations in the *MICU1* gene causing myopathy with extrapyramidal signs (MPXPS) is an autosomal recessive disorder characterized by the early onset of proximal muscle weakness, motor disorders, and learning difficulties in children, with most patients developing progressive extrapyramidal signs, potentially leading to disability. (2) There are also reports of a patient with brain MRI showing congenital malformations, including polymicrogyria and cerebellar hypoplasia, (3) which is consistent with the characteristic of the four children in this study showing more significant muscle weakness and motor disorders with increasing age.

In this case, we identified compound heterozygous mutations c.156G > A and c.235G > T, loss1 and c.1,372C > T in two families, which have not been reported in previous cases, and their corresponding clinical features, while having some similarities with previously reported MPXPS, still differ due to different *MICU1* gene mutation sites. Interestingly, the three point mutations identified in this study are all nonsense mutations, but in theory, the c.1,372C > T variant, located in the last exon, may lead to the production of a truncated mutant *MICU1* protein (Figure 2), which is consistent with its “uncertain pathogenicity” classification and relatively low evolutionary conservation. It is speculated that the two patients in Family 2 may have different phenotypes from the two patients in Family 1, and whether the phenotype is relatively light or severe depends on whether the c.1,372C > T mutation causes a mild loss of function (LoF) consequence or a more severe gain of function (GoF) effect.

The two brothers in Family 1 carrying compound heterozygous mutations c.156G > A and c.235G > T exhibited distinct clinical manifestations. The elder brother presented with early-onset muscle weakness and motor impairment, followed by the progressive development of attention deficit, hyperactivity disorder, and learning difficulties, which are consistent with the typical clinical features of MPXPS. Although the younger brother currently remains asymptomatic, he may develop similar symptoms as his elder brother with advancing age. Furthermore, despite the absence of extrapyramidal signs and symptoms in both brothers at present, close clinical monitoring is warranted as they may potentially develop extrapyramidal manifestations during disease progression. The two sisters in Family 2 identified with compound heterozygous mutations EXON4-8 loss and c.1,372C > T, the older sister showed early flat feet, retrosternal pain, hyperactivity, and poor concentration, but no muscle weakness, motor disorders, or extrapyramidal symptoms, and the younger sister has no clinical manifestations.

The common point of the four children in this study is the early detection of elevated muscle enzymes and transaminase abnormalities, and after treatment with levocarnitine, Glutathione, and compound glycyrrhizin tablets, the muscle enzymes and transaminase did not improve significantly and remained abnormal. Combining all reported cases of MPXPS caused by *MICU1* gene variants, there is persistent elevation of muscle enzymes and transaminase abnormalities, so in clinical practice, early detection of persistent elevation of muscle enzymes and transaminase abnormalities, especially in families with similar patients, in addition to considering progressive muscular dystrophy, MPXPS should also be vigilant. In addition, the pathogenic mechanism of the *MICU1* gene c.1,372C > T variant is worth further basic research.

## Consent statement

The studies involving humans were approved by Institutional Review Board at Children’s Hospital of Chongqing Medical University. The studies were conducted in accordance with the local legislation and institutional requirements. Written informed consent for participation in this study was provided by the participants’ legal guardians/next of kin. Written informed consent was obtained from the individual(s), and minor(s)’ legal guardian/next of kin, for the publication of any potentially identifiable images or data included in this article.

## Data Availability

The datasets presented in this study can be found in online repositories. The names of the repository/repositories and accession number(s) can be found in the article/supplementary material.
